# miR-TRAP: A Benchtop Chemical Biology Strategy to Identify microRNA Targets**

**DOI:** 10.1002/anie.201201512

**Published:** 2012-05-08

**Authors:** Huricha Baigude, Zhonghan Li, Ying Zhou, Tariq M Rana

**Affiliations:** Program for RNA Biology, Sanford–Burnham Medical Research Institute10901 N Torrey Pines Road, La Jolla, CA 92037 (USA)

**Keywords:** gene silencing, microRNA, mRNA, psoralen, noncoding RNAs

microRNAs (miRNAs) are single-stranded small RNAs consisting of approximately 21 nucleotides that regulate posttranscriptional gene expression in metazoans and plants. miRNAs are usually generated from noncoding regions of gene transcripts and function to suppress gene expression by translational repression or RNA degradation. In recent years, miRNAs have been shown to be regulators of numerous activities, including developmental processes, disease pathogenesis, and host–pathogen interactions.^[1]^ Regulation of genes by miRNAs is a wide-spread phenomenon, and according to recent miRNA annotation and deep-sequencing data, there are >15 000 microRNA gene loci spanning >140 species and >17 000 distinct mature microRNA sequences.^[2]^ These numbers will surely increase as high-throughput RNA sequencing technologies are applied to the discovery of new noncoding RNAs.

The major biogenesis pathway of mature miRNAs requires digestion of the precursor RNA hairpin structure by two members of the RNase III family, Drosha and Dicer, while other miRNAs are generated through splicing of miR-coding introns.^[1]^, ^[3]^ Processed miRNAs are loaded into the Argonaute (Ago) protein assembly, known as the RNA-induced silencing complex (RISC), which is the catalytic engine for miRNA-mediated posttranscriptional regulation. In general, miRNAs bind (with some mismatches) to the 3′ untranslated region (UTR) of target mRNAs and block their expression by directly inhibiting translation and/or destabilizing the mRNA.^[4]^

Defining functional mRNA targets of a specific miRNA is crucial to understand RNA-based mechanisms regulating cell growth, development, differentiation, and disease processes. miRNA targets can be computationally predicted using basic rules that have been discovered by previous functional studies.^[5]^ For example, four popular algorithms, including Targetscan,^[6]^ miRanda,^[7]^ miRbase,^[8]^ and PicTar,^[9]^ predict miRNA targets in mRNAs based on complementary seed sequences, 2–7 nucleotides from the 5′ end of the miRNA guide strand. By analyzing RISC-associated mRNA transcripts, the computational method mirWIP was developed to predict miRNA targets in *C.* *elegans.*^[10]^ These computational tools are useful to predict possible miRNA targets for further experimental validation. However, the typical number of candidate target mRNAs for a given miRNA is large and may vary depending on the algorithm used for prediction, making experimental validation difficult and time consuming. Therefore, efficient, reliable, and straightforward experimental approaches are greatly needed to identify and validate predicted miRNA targets.

Recently, immunoprecipitation of native Ago protein crosslinked to mouse brain RNA, followed by high-throughput sequencing, and bioinformatic analysis (a technique known as HITS-CLIP) has been used to identify miRNA targets.^[11]^ To overcome low RNA–protein crosslinking efficiency by exposure to 254 nm UV radiation and to reduce nonspecific RNA crosslinking, an improved method, named PAR-CLIP (photoactivatable-ribonucleoside-enhanced crosslinking and immunoprecipitation), was developed to isolate and identify RNA regions bound by RNA-binding proteins, including Ago2.^[12]^ These approaches have provided a wealth of molecular information regarding RNA–protein assemblies as well as miRNA targets identified in Ago2 complexes. However, these approaches require immunoprecipitation of an RNA–Ago2 complex, purification of the crosslinked product, cDNA library preparation, PCR amplification, deep sequencing, and finally bioinformatic analysis to provide a list of miRNA targets for experimental validation. The process is time-consuming, can be costly, and requires access to sophisticated deep sequencing and bioinformatic facilities. Therefore, a straightforward benchtop method is needed to directly identify miRNA targets.

To develop such a method, we conjugated psoralen (Pso) to miRNAs, to produce highly photoreactive probes. Psoralen analogues have been used extensively to study nucleic acid structure and function, in vitro and in vivo.^[13]^ One advantage of using Pso for photocrosslinking is that the reaction uses longer wavelength UV radiation (360 nm), which is less harmful to cells than the 254 nm UV radiation used for HITS-CLIP, making it preferable for in vivo studies. Moreover, the Pso-crosslinked product can be photoreversed by treatment with short wave UV radiation (254 nm).^[14]^

We named our strategy miRNA target RNA affinity purification (miR-TRAP). Our goals in developing miR-TRAP technology were threefold. First, the method should serve to directly identify miRNA targets in vivo. Second, the method should not only identify mRNA targets whose expression is down regulated by miRNAs but also those mRNAs whose translation is repressed; such targets are not cleaved by RISC and cannot be identified by quantitative PCR and microarray analysis. Third, our goal was to eliminate the use of antibodies, which can create nonspecific background signals and complicate data interpretation. The reason we chose to use Pso-modified miRNA mimics as probes for target identification is that this type of probe functions similarly to endogenous miRNAs; a Pso-modified miRNA guide strand incorporated into RISC finds and then binds a specific target mRNA.^15^ When cells are exposed to UVA radiation (360 nm), the Pso moiety on the miRNA mimic reacts with uridine on target mRNAs,^[13b]^ enabling the covalently bound complex to be easily pulled down by biotin-streptavidin affinity purification. Such a crosslinking and pulldown method could significantly enrich a population for a specific target sequence, which could then be quickly analyzed by quantitative PCR.

To create functional photoreactive miRNA probes, we chemically modified miRNA mimics at distinct positions in the antisense/guide strand. Pso was covalently attached to a uridine residue 3′ of the miRNA seed sequence (position 9 from the 5′ end), while a biotin was conjugated at the 3′ end as an affinity tag (see Supporting Information for sequences). We first synthesized activated *N*-hydroxysuccinimide esters of psoralen with different linkers (Supporting Information, Scheme S1). Activated Pso was then conjugated to amine-modified miRNA mimics at either position 5 of the uridine (designated as 5-S-Pso, or 5-l-Pso) or the 2’ position of the sugar ring of a uridine (designated as 2′-S-Pso, or 2′-l-Pso; Figure 1 a), with either short (S) or long (L) linkers. We chose miRNA-29a and miRNA-135b to establish our method, since both these miRNAs function in many cellular pathways, such as reprogramming of mouse embryonic fibroblasts (MEFs) into induced pluripotent stem (iPS) cells.^[1e]^, ^[1f]^ To ensure a higher yield of Pso-modified miRNAs, the coupling reaction was conducted using an excess molar ratio of activated Pso to amine-containing miRNA mimics (2′O protected, Dharmacon) in DMSO in the presence of *N*,*N*-diisopropylethylamine (DIPEA) in the dark. After 24 h, excess Pso was removed by precipitating the modified miRNAs with ethyl acetate, and the Pso-functionalized miRNA mimics were analyzed by UV spectroscopy and denaturing polyacrylamide gel electrophoresis. Compared with the unmodified miRNA mimic, Pso-modified miRNA showed a distinct shift in absorption in the UV spectrum (from 260 nm to 255 nm) and increased absorption at 310 nm, indicative of Pso conjugation to the RNA strand (data not shown). Subsequent analysis of deprotected single-stranded RNAs by denaturing gel electrophoresis clearly showed single bands with slower electrophoretic mobility compared to the unmodified strand, confirming that the miRNAs were efficiently conjugated to Pso (Figure 1 b).

**Figure 1:**
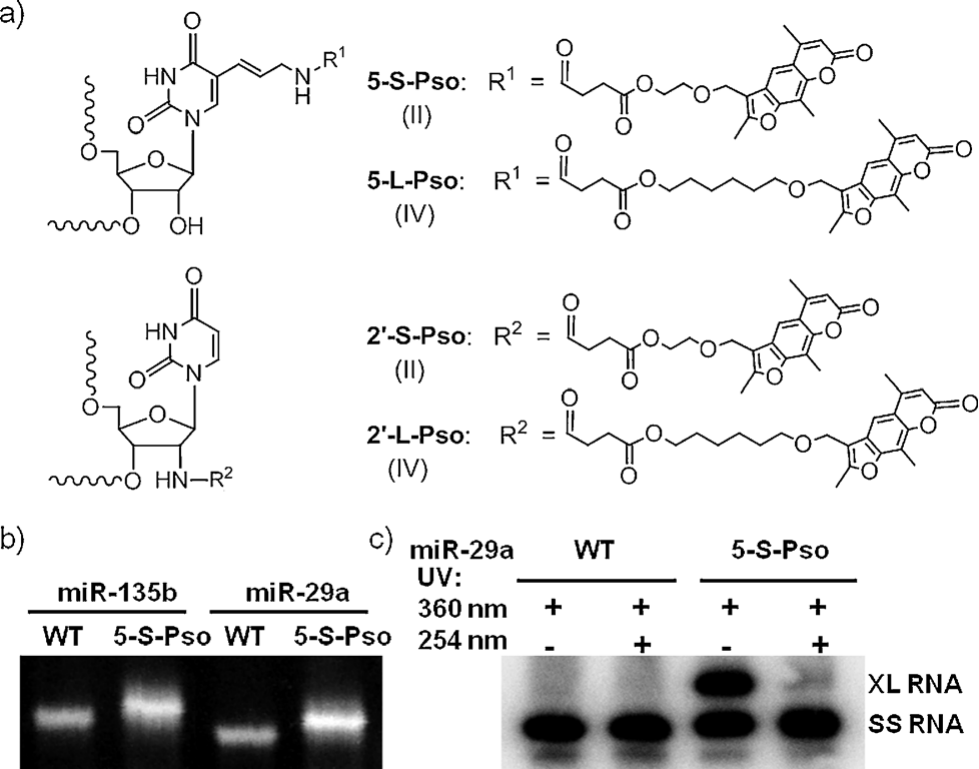
a) Structures of psoralen-modified uridine. Psoralen with different linkers (S or L) was conjugated to uridine, nine nucleotides from the 5′ end of miRNAs. b) RNA conjugation with psoralen. Gel electrophoresis of deprotected, single-stranded unmodified and Pso-modified miRNAs. c) In vivo RNA photocrosslinking. After transfection with miRNAs, MEF cells were exposed to long wave UV radiation (360 nm) to induce crosslinking followed by UV radiation (254 nm) to photoreverse the Pso-crosslinked product. RNAs pulled down by streptavidin beads were radiolabled with ^32^P phosphate and separated by denaturing gel electrophoresis. A crosslinked RNA duplex (XL RNA) with slower electophoretic mobility than single-stranded RNA (SS RNA) is shown.

We hypothesized that the Pso-conjugated antisense/guide strand of a miRNA mimic duplex should react with the complementary sense/passenger strand upon UVA treatment and that crosslinked RNA should show a distinct shift in mobility under gel electrophoresis. Moreover, because Pso crosslinking is photoreversible, the slower band should disappear when miRNA was treated with UVB (254 nm). Indeed, a slow-moving, photoreversible band was observed only when the Pso-modified miRNA mimic was treated with UVA (360 nm), confirming the efficiency of crosslinking and the identity of the crosslinked strands (Supporting Information, Figure S1).

Next we investigated the in vivo photoreactivity of Pso-modified miRNA probes using two approaches: 1) crosslinking between antisense/guide and sense/passenger strands in cells, and 2) more significantly, crosslinking of the antisense strand in RISC with endogenous mRNAs. We transfected MEFs with a double stranded miRNA-29a mimic either with or without Pso conjugation. After 48 h, cells were exposed to UVA for 5 min at room temperature and total RNA was extracted. Half of the RNA was used to pull down biotinylated RNA using streptavidin beads, and the other half was run on denaturing agarose gels, transferred to membranes, and probed using a commercial biotin detection kit to assess crosslinking between miRNAs and large cellular RNAs. The streptavidin pulldown portion of total RNA was radiolabeled with ^32^P phosphate and analyzed on a 14 % denaturing gel for small RNAs. An RNA-crosslinked (XL RNA), slower mobility band of Pso-modified miR-29a was observed when cells were exposed to 360 nm UV radiation, a reaction readily reversed by 254 nm UV radiation, demonstrating that miRNA probes can be photocrosslinked with high efficiency (Figure 1 c). Pso-modified miRNAs not only crosslink to their complementary strand but also crosslink to endogenous RNAs when loaded into RISC; a population of large, crosslinked RNAs was isolated from cells transfected with Pso-conjugated miRNA-29a but not with unmodified miRNA-29a, indicating that 5-S-Pso was specifically able to form crosslinks with target RNAs (Supporting Information, Figure S2).

Cellular function of miRNAs can be impaired by chemical modification. To confirm the functionality of Pso-modified miRNAs, we transfected HeLa cells with a luciferase reporter vector containing a perfectly matched site for miRNA-29a in the 3′UTR of the firefly luciferase gene. The Pso-modified miRNA-29a mimic silenced luciferase gene expression with an efficiency similar to an unmodified miRNA mimic, indicating that Pso-modified miRNA-29a is completely functional (Figure 2 a). Pso-modified miRNA-29a function was further evaluated using a second luciferase assay with a vector expressing a 3′UTR from the HIV genome, which we previously identified as a miRNA-29a target.^[16]^ Again, luciferase expression levels decreased in cells transfected with Pso-modified miRNA-29a, similar to the effect seen with unmodified miRNA-29a (Supporting Information, Figure S3).

**Figure 2:**
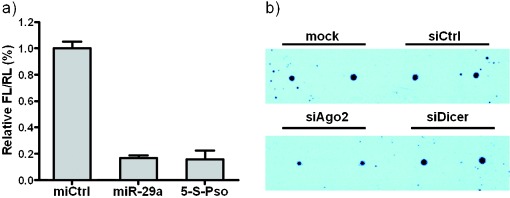
a) Psoralen-modified miRNA is functional. miRNA-29a (unmodified or modified with psoralen) was transfected into cells together with a plasmid expressing firefly luciferase containing a perfectly matched miRNA-29a target site in the 3′UTR. Firefly/Renilla luciferase (FL/RL) ratios are normalized to those of control miRNAs. b) The biotin signal from Pso-miRNA crosslinked to mRNA is significantly decreased following Ago2 knockdown. MEFs were transfected with transfection agent alone (mock), a control siRNA (siCtrl), siAgo2, or siDicer. After 24 h, all cells were transfected with miRNA-29a (5-S-Pso). Two days later cells were exposed to UVA for 5 min, and total cellular mRNA was isolated using oligo(dT) beads. 30 ng polyA RNA was blotted onto a membrane, and the biotin content of the RNA was detected using a BrightStar BioDetection kit (Ambion).

Photoreaction between Pso-modified miRNAs and large cellular RNAs could occur nonspecifically. To determine whether crosslinking resulted from interaction of miRNA and specific mRNA targets, we used siRNAs to knock down either Ago2 or Dicer in cells and monitored changes in crosslinking efficiency. Ago2 is the key component of the RISC complex, whose function is required for RNAi activity, while Dicer has less effect on RNAi activity, including the action of both siRNAs and miRNAs.^[17]^ Indeed, Ago2 knockdown decreased biotin signal intensity in total cellular mRNA (indicating a decrease in crosslinking to mRNAs), whereas no change was observed when Dicer was knocked down (Supporting Information, Figure S4, Figure 2 b). These results demonstrate that a Pso-modified miRNA mimic is assembled into RISC and becomes specifically crosslinked to mRNA targets through the RISC complex upon UVA treatment.

We next identified specific miRNA-135b and miRNA-29a targets in MEFs. Pso-modified miRNAs were transfected into MEFs and 24 h later cells were treated with UVA (360 nm) for 5 min at room temperature. Cells were lysed in a lysis buffer containing an RNase inhibitor, and biotinylated RNAs were affinity purified using streptavidin magnetic beads. To establish assay efficiency and specificity without performing microarray analysis or deep sequencing, we randomly chose predicted target genes for each miRNA from TargetScan and performed RT-qPCR analysis on affinity purified RNAs. Compared with the negative control, Tet2 RNA was enriched 20-fold in pulldowns from cells transfected with Pso-modified miRNA-29a. Pulldown of Pso-modified miRNA-135b gave approximately 4.5-fold enrichment of one of its predicted targets, Elk3 (Figure 3). Further investigation of crosslinking efficiency of miR-29a conjugated with Pso through different linkers (S or L) showed that shorter linkers are more efficient than longer ones (data not shown). By using 5-S-Pso and 2′-S-Pso modified miR-29a, we identified additional targets of miR-29a in MEFs (Supporting Information, Figure S5).

**Figure 3:**
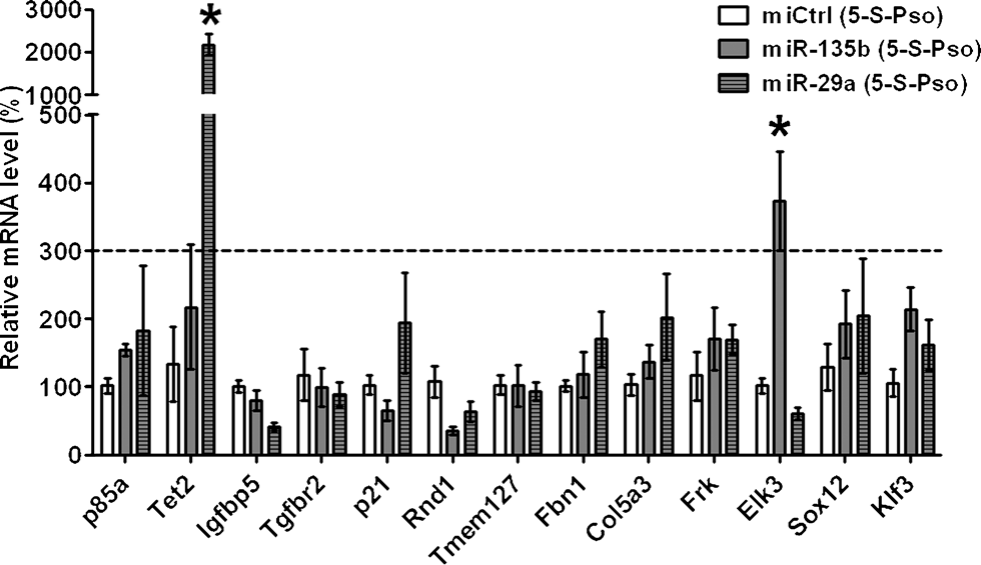
Enrichment analysis of pulldown RNA from cells transfected with Pso-modified miRNAs: a nontargeting control (miCtrl), miRNA-29a, or miRNA-135b. A total of 13 genes were randomly selected from a list of predicted miRNA-29a and miRNA-135b targets in TargetScan and analyzed by RT-qPCR. GAPDH RNA levels served as an internal control. The average of three independent experiments is expressed as the mean±SEM (standard error of the mean), relative to control.

To validate whether the enriched mRNAs are genuine miRNA targets, we cloned the 3′UTRs of Tet2 and Elk3 into the luciferase-expressing pGL3 vector, as representative miRNA-29a and miRNA-135b targets, respectively, and expressed them in HeLa cells as described previously.^[18]^ Strong inhibition of luciferase expression was detected when the miRNA-135b mimic was co-transfected with pGL3 containing the Elk3 3′UTR, confirming that Elk3 is a target of miRNA-135b in MEFs. Similarly, a miRNA-29a mimic significantly decreased luciferase activity in cells expressing the Tet2 3′UTR, confirming it as a miRNA-29a target (Supporting Information, Figure S6).

In conclusion, we have established a unique method to efficiently identify miRNA targets by simple RT-qPCR by conjugating psoralen to a miRNA and performing a long wave UV photocrosslinking reaction. Nonspecific binding between cellular RNA and streptavidin beads was minimized by use of lysis buffer containing 0.5 % of the detergent NP-40 followed by stringent washing steps during the pulldown procedure. In fact, total poly(A)-containing RNAs from cells transfected with Pso-modified miRNAs but not exposed to UVA did not exhibit any biotin signal (data not shown), suggesting that RNAs from cells exposed to UVA were indeed covalently crosslinked and formed biotinylated miRNA/mRNA complexes. Analysis of pulldown RNA by RT-qPCR for only 13 predicted targets identified two novel targets, Elk3 for miRNA-135b and Tet2 for miRNA-29a.^[19]^ Remarkably, we observed approximately 4–20-fold enrichment of miRNA targets. We are now applying these methods to identify miRNA targets in various disease models.

## Experimental Section

Photocrosslinking and target analysis: MEFs (CF1) were prepared according to previously reported methods.^[1e]^[1e], [1f] MEFs were cultured in DMEM medium (Invitrogen) with 10 % FBS (fetal bovine serum; Invitrogen) plus glutamine and nonessential amino acids (NEAA). For crosslinking experiments, MEFs were seeded in 15 cm dishes at a density of 2.1×10^6^ cells/dish. MEFs were transfected with Pso-modified miRNA negative control, miRNA-135b, or miRNA-29a, at a final concentration of 25 nM using Lipofectamine 2000 (Invitrogen), according to the manufacturer’s protocol. Twenty-four hours later, the culture medium was removed and cells were washed three times with PBS buffer, pH 7.4. PBS (10 mL) was added and the cells were treated for 5 min with UVA (360 nm) in a photochemical reactor (RAYNOET, model RPR-100) equipped with 16 RPR-3500 light tubes. Cells were then lysed in lysis buffer (3.0 mL; 20 mM Tris, 200 mM NaCl, 2.5 mM MgCl_2_, 0.5 % NP-40, pH 7.5, 80 U RNaseOUT and freshly added protease inhibitor cocktail) and shaken on a rocker at 4 °C for 5 min. Cells were collected by scraping the plates, and cell debris was removed by centrifugation at 13 000 rpm at 4 °C for 15 min. The supernatant was used for pulldown of miRNA targets. Dynabeads M-280 Streptavidin (50 µL; Invitrogen) were mixed with cell lysis supernatant (ca. 1.0 mL) and rotated at 4 °C for 4 h. The supernatant was removed after placing the tubes on a magnet for 2 min, and the beads were washed with lysis buffer (three times with 500 µL, 5 min each time). To release RNA from the beads, RNase-free water (50 µL) and TRIzol reagent (200 µL) were added to the beads, and RNA was extracted according to the manufacturer′s protocol (Invitrogen). Final RNA samples were suspended in 7.5 µL RNase-free water and used for reverse transcription reactions using Superscript II (Invitrogen). qPCR was performed using a Roche LightCycler480 II and a Sybr green mixture from Abgene (Ab-4166). Primers used in qPCR are listed in Supporting Information, Table S1.

## Notes

Detailed facts of importance to specialist readers are published as ”Supporting Information”. Such documents are peer-reviewed, but not copy-edited or typeset. They are made available as submitted by the authors.
