# Supplementary material for: miR-TRAP: A Benchtop Chemical Biology Strategy to Identify microRNA Targets
Source: Angew Chem Int Ed Engl. 2012 May 8;51(24):5880–3. doi: 10.1002/anie.201201512 (PMC3501383; doi:10.1002/anie.201201512)

Angewandte Chemie
Eine Zeitschrift der Gesellschaft Deutscher Chemiker

Supporting Information

© Wiley-VCH 2012

69451 Weinheim, Germany

miR-TRAP: A Benchtop Chemical Biology Strategy to Identify microRNA Targets**

*Huricha Baigude, Ahsanullah, Zhonghan Li, Ying Zhou, and Tariq M. Rana**

anie_201201512_sm_miscellaneous_information.pdf

Materials and Methods

4'-(2-Hydroxyethoxy)methyl-4,5',8-trimethylpsoralen was purchased from Berry & Associates. Succinic anhydride, N-(3-Dimethylaminopropyl)-N'-ethylcarbodiimide (EDCI), N-hydroxylsuccinimide (NHS), N,N-diisopropylethylamine (DIPEA) and dimethyl sulfoxide (DMSO) were purchased from Sigma Aldrich. 1H NMR and 13C NMR were recorded on a JEOL Alpha-400 NMR spectrometer. Chemical shifts are reported in ppm and referenced to the corresponding residual nuclei in deuterated solvents.

Synthesis of activated Psoralen derivatives

Compound II. 4'-(2-Hydroxyethoxy)methyl-4,5',8-trimethylpsoralen (40mg), succinic anhydride (15mg) and catalytic amount of dimethylaminopyridine (DMAP) were dissolved in dry THF and stirred at r.t. overnight. Then *N*-hydroxysuccinimide (NHS) and EDCI were added and stirring was continued additional 5 hr. The reaction mixture was applied to a silica gel column and final product was purified by eluting with DCM and methanol (15:1).^{1}H NMR (400 MHz, CDCl3): δ 7.53 (s, 1H), 6.17 (s, 1H), 4.60 (s, 2H), 4.22 (t, J = 4.6, 2H), 3.60 (t, J = 4.8, 2H), 2.82 (t, J = 6.8, 2H), 2.76 (s, 4H), 2.63 (t, J = 7.3, 2H), 2.49 (s, 3H), 2.42 (d, 6H).^{13}C NMR (400 MHz, CDCl3): δ 170.8, 168.8, 167.5, 161.5, 155.1, 154.6, 153.3, 124.9, 116.2, 112.8, 111.5, 109.1, 67.2, 64.0, 63.1, 28.5, 26.1, 25.5, 19.3, 12.2, 8.4. ESI-MS (*m/z*): $[M]^+$ calcd for C25H25NO10 499.15; found, 500.1.

Compound IV. Compound IV was synthesized in the similar way with spacer 1 using 4'-(6-Hydroxyhexyloxy)methyl-4,5',8-trimethylpsoralen as starting material. ^{1}H NMR (400 MHz, CDCl3): δ 7.60 (s, 1H, arom.), 6.24 (s, 1H, alkene), 4.62 (s, 2H, CH_2), 4.08 (t, J = 6.6, 2H, CH_2),

3.47 (t, J = 6.6, 2H, CH_2), 2.94 (t, J = 7.3, 2H, CH_2), 2.82 (s, 4H, anhydride), 2.72 (t, J = 7.5, 2H, CH_2), 2.58 (s, 3H, CH_3), 2.49 (d, 6H, $2CH_3$), 1.25-1.63 (2m, 8H, $4CH_2$). ^{13}C NMR (400 MHz, CDCl3): δ 172.4, 170.9, 169.7, 168.8, 167.7, 161.5, 154.6, 153.3, 149.1, 125.1, 116.1, 112.7, 111.6, 109.0, 69.9, 65.0, 63.0, 52.0, 37.3, 33.5, 29.5, 28.6, 26.2, 25.5, 19.3, 12.3, 8.4. ESI-MS (*m*/*z*): $[M+Na]^+$ calcd for C29H33NO10, 578.20; found, 578.24.

Modification of microRNA mimics

microRNA mimics were purchased from Dharmacon. The sequences are:

cel-miRNA-67 (non-targeting control):

Antisense/guide strand: 5'P-UCACAACC(5-N-U)CCUAGAAAGAGUAGA-Biotin-3'

Sense/passenger strand: 5'UCUACUCUUUCUAGGAGGUUGUGA-3'

mmu-miRNA-135b:

Antisense/guide strand: 5'P-UAUGGCUU(5-N-U)UCAUUCCUAUGUGA-Biotin 3'

Sense/passenger strand: 5'P-UCACAUAGGAAUGAAAAGCCAUA 3'

mmu-miRNA-29a:

For miR-29a (5-S-Pso) and miR-29a (5-L-Pso):

Antisense/guide strand: 5' P-UAGCACCA(5-N-U)CUGAAAUCGGUUA-Biotin 3'

For miR-29a (2'-S-Pso) and miR-29a (2'-L-Pso)

Antisense/guide strand: 5' P-UAGCACCA(2'-N-U)CUGAAAUCGGUUA-Biotin 3'

Sense/passenger strand: 5' P-UAACCGAUUUCAGAUGGUGCUA 3'

The 2'-ACE protected antisense/guide strand microRNA mimic was dissolved in DMSO. Activated Psoralen derivatives (compound II or IV) was added (1:50 molar ratio) and the reaction mixture was incubated at r.t. for 24 h. Ethyl acetate was then added, and the precipitate

was collected by centrifugation. The pellet was resuspended in ethyl acetate and repeatedly washed with ethyl acetate. After air-drying the pellet, 2'-deprotection buffer (provided by Dharmacon) was added, followed by heating at 65 ^{o}C for 2 h. For annealing, equimolar amount of deprotected antisense/guide strand and the sense/passenger strand were mixed and heated at 60 ^{o}C for 10 min then incubated at r.t. for 30min.

Specificity of Pso-mediated crosslinking

MEFs were seeded at a density of 1.5 x10^{5}/well in 6-well plate. An siRNA negative control or siAgo2 or siDicer (Dharmacon) was transfected at 50 nM final concentration using Lipofectamine 2000 (Invitrogen). The next day Ps-modified miRNA mimics were transfected at 50 nM final concentration into previously transfected cells. After 48 h, total RNA was extracted with Trizol reagent (Invitrogen), and 350 ng RNA was used for RT and subsequent qPCR to analyze Ago and Dicer mRNA levels. The remaining RNA was used for poly(A) RNA purification using Dynabeads Oligo (dT)25 (Invitrogen), according to the manufacturer's instruction. PolyA RNA (25 ng) was spotted on a membrane and blotted for biotin detection using a BrightStar BioDetect kit (Ambion).

Dual luciferase assay

The respective 3′UTRs of Tet2 and Elk3 were cloned into the *Xba*I site of pGL3 control vectors. 1×10^{5} HeLa cells were seeded in 12-well plates. After 24 h, miRNAs (50 nM) together with 200 ng of the reporter vectors and 75 ng of pRL-TK (renilla luciferase) were transfected (Lipofectamine 2000, Invitrogen), and cell lysates were harvested at day 2 post-transfection. 20 µl of lysates were then used for a dual luciferase assay (Dual-Luciferase Reporter Assay System Promega, E1910) following the manufacturer's protocol.

Supplementary Figure Legends

Supplementary Scheme 1. Synthesis of activated Psoralen derivative **II** and **IV**. a) 4'-(2-hydroxyethoxy)methyl-4,5',8-trimethylpsoralen (I), or 4'-(6-hydroxyhexyloxy)methyl-4,5',8-trimethylpsoralen (III), succinic anhydride (1.2 equiv), DMAP, THF, r.t. 2 h, yield: 83%; b) NHS (1.2 equiv), EDCI, THF, r.t. 5 h, yield: 65%. DMAP=dimethylaminopyridine, NHS=N-hydroxysuccinimide, THF=tetrahydrofuran, EDCI=N-(3-Dimethylaminopropyl)-N'-ethylcarbodiimide.

Supplementary Figure 1. Photo reactivity of Psoralen modified miRNA-29a. Antisense/guide (GS), sense/passenger (PS), and Psoralen modified duplex miRNA-29a (GS/PS) with and without UVA and/or UVB treatment were analysed by denaturing gel electrophoresis.

Supplementary Figure 2. Crosslinking of Psoralen modified miRNA-29a with endogenous RNAs. After transfection with miRNAs, MEF cells were exposed to long wave UV (360 nm) to induce crosslinking. 30 ug of total RNA from MEFs transfected with unmodified miRNA-29a or Psoralen-modified miRNA-29a was analyzed on a 1% denaturing agarose gel. RNA was transferred to a membrane and probed for biotin signal using a BrightStar BioDetection kit (Ambion). An enhanced signal from biotin-conjugated large cellular RNA was observed only in total RNA extracted from Pso-modified miRNA-29a-transfected MEFs

Supplementary Figure 3. Psoralen modified miRNA mimics are functional. miRNA 29a (unmodified or modified with Psoralen) was transfected into cells with a plasmid expressing firefly luciferase containing HIV-1 3' UTR. Psoralen-modified miRNA inhibited luciferase

expression with efficiencies similar to those seen with unmodified miRNA. Firefly/Renilla (FL/RL) ratios are normalized to those of control miRNAs.

Supplementary Figure 4. qPCR analysis of Ago2 and Dicer mRNA level 48 h after siRNA transfection. GAPDH served as internal control. The average of three independent experiments is expressed as the mean±SEM relative to control.

Supplementary Figure 5. Enrichment analysis of pulldown RNA from MEF cells transfected with Pso-modified miRNA-29a (5-S-Pso and 2'-S-Pso) compared to nontargeting control (miCtrl, 5-S-Pso). GAPDH served as internal control. The average of three independent experiments is expressed as the mean±SEM relative to control.

Supplementary Figure 6. Validation of miRNA targets by dual luciferase assays in HeLa cells. Luciferase assays were performed by cloning the 3'UTR of either Tet2 or Elk3 into the pGL3 vector as representative targets of miRNA-29a or miRNA-135b, respectively. Firefly and Renilla (FL and RL) luciferase expression was analyzed by co-transfecting cells with miRNAs and expression vectors as described previously [18]. Firefly/Renilla (FL/RL) ratios are normalized to those of control miRNAs.

Supplementary Table 1. List of primers used for RT-qPCR.

**Name**	**Sequence**
mmuGapdh_F	5’-atcaagaaggtggtgaagcggaa-3’
mmuGapdh_R	5’-tggaagagtgggagttgctgttga-3’
mmuP85a_F	5’-agtggaagggtccttagcaagcat-3’
mmuP85a_R	5’-agaggttctcgatgaaggccagtt-3
mmuTet2_F	5’-gtggactgcgaggctgag-3’
mmuTet2_R	5’-agtcttgggagggcaagc-3’
mmuIgfbp5_F	5’-ggcgagcaaaccaagataga-3’
mmuIgfbp5_R	5’-aggtctcttcagccatctcg-3’
mmuTgfbr2_F	5’-Agaagccgcatgaagtctg-3’
mmuTgfbr2_R	5’-ggcaaaccgtctccagagta-3’
mmuP21_F	5'-tccacagcgatatccagaca-3'
mmuP21_R	5'-ggacatcaccaggattggac-3'
mmuRnd1_F	5’-ggtgctagcgaaagactgcta-3’
mmuRnd1_R	5’-tgtcccagagactaagctcca-3’
mmuTmem127_F	5’-tgtaaacccggatctgctaaa-3’
mmuTmem127_R	5’-aagcagaaagcagcgatgac-3’
mmuFbn1_F	5’-gcgctaatgccagaccag-3’
mmuFbn1_R	5’-tccccaggtatgtttgtgc-3’
mmuCol5a3_F	5’-tgatgggaacccaggaca-3’

mmuCol5a3_R	5'-acaagcagctcctggatgtct-3'
mmuFrk_F	5'-aacaagtgacgggctgtgt-3'
mmuFrk_R	5'-ggggttggtacctggatctt-3'
mmuElk3_F	5'-gagcagccttagtcctgtcg-3'
mmuElk3_R	5'-gttgagcagtgtggggaact-3'
mmuSox12_F	5'-cagtcctgtcccgcgtag-3'
mmuSox12_R	5'-ggtatgtaggaggaacgaggtg-3'
mmuGlis2_F	5'-tgccccacctgtaacaaga-3'
mmuGlis2_R	5'-cagacgtagggcttctcacc-3'
mmuKlf3_F	5'-tcgcacttgaaagcacaca-3'
mmuKlf3_R	5'-tcccaggtgcatttgtacg-3'
mmuTet1_F	5'-GGC TCC AGT TGC TTA TCA AAA-3'
mmuTet1_R	5'-CCC TCT TCA TTT CCA AGT-3'
mmuTet3_F	5'-GCC TCA CGG GAG ACA ATC-3'
mmuTet3_R	5'-TGG CCA GAT CCT GAA AGC TA-3'
mmuTdg_F	5'-TTG TGG CAT TGC TTC AAA TG-3'
mmuTdg_R	5'-CTG CCC ATT CGG AAC ATC-3'
mmuSept8_F	5'-GAG GCC CTG CAG TCA CAG-3'
mmuSept8_R	5'-AGG GAA CTC GCT TCA GTT TG-3'
mmuDtwd2_F	5'-CAC TCC AGA TCT CTA CCC ACT TGT-3'
mmuDtwd2_R	5'-GAC TGT GCG CAA CAC TCT G-3'
mmuNasp_F	5'-TGG GTG ACA TTC CAG CAG-3'
mmuNasp_R	5'-GCC GTT TCT CCA TAC TTC TTA CC-3'

mmuAgo2_F	5'-gcgtcaacaacatcctgct-3'
mmuAgo2_R	5’-ctcccaggaagatgacaggt-3’
mmuDicer1_F	5’-gggctgtatgagagattgctgatg-3’
mmuDicer1_R	5’-cacggcagtctgagaggatttg-3’

References

1. a) Z. Li, C. S. Yang, K. Nakashima, T. M. Rana, *EMBO J* **2011**, *30*, 823-834; b) C. S. Yang, Z. Li, T. M. Rana, *RNA* **2011**, *17*, 1451-1460.

Supplementary Scheme 1

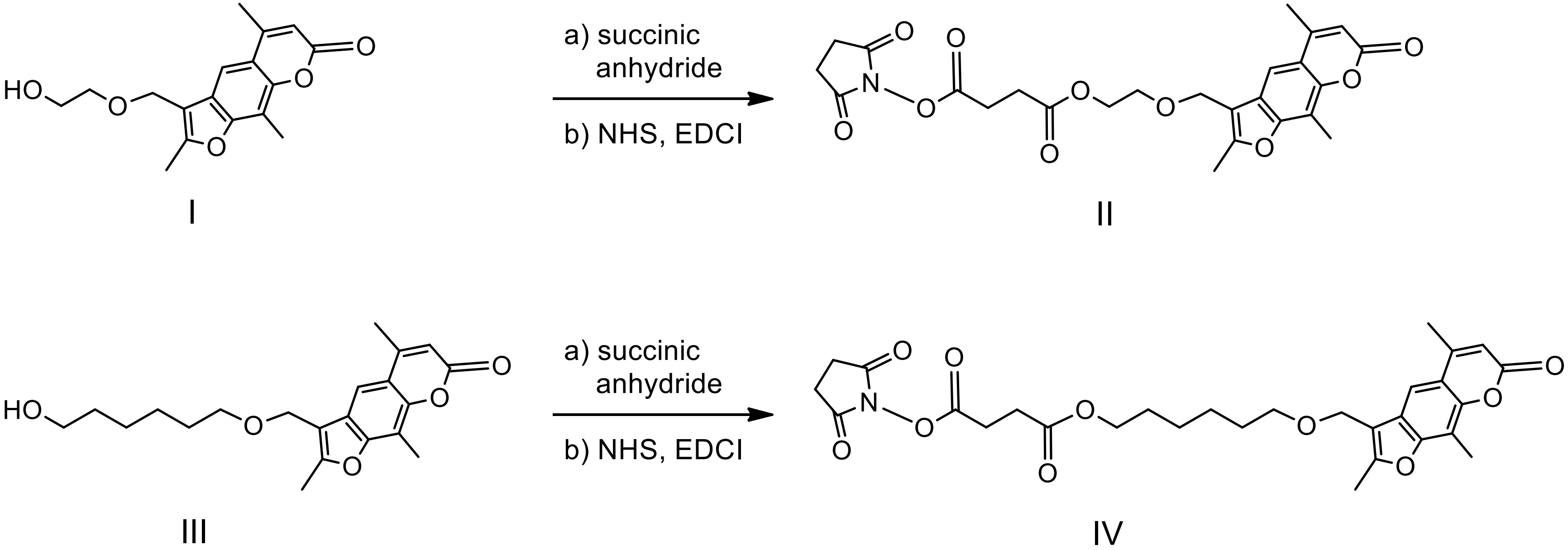

Supplementary Figure 1

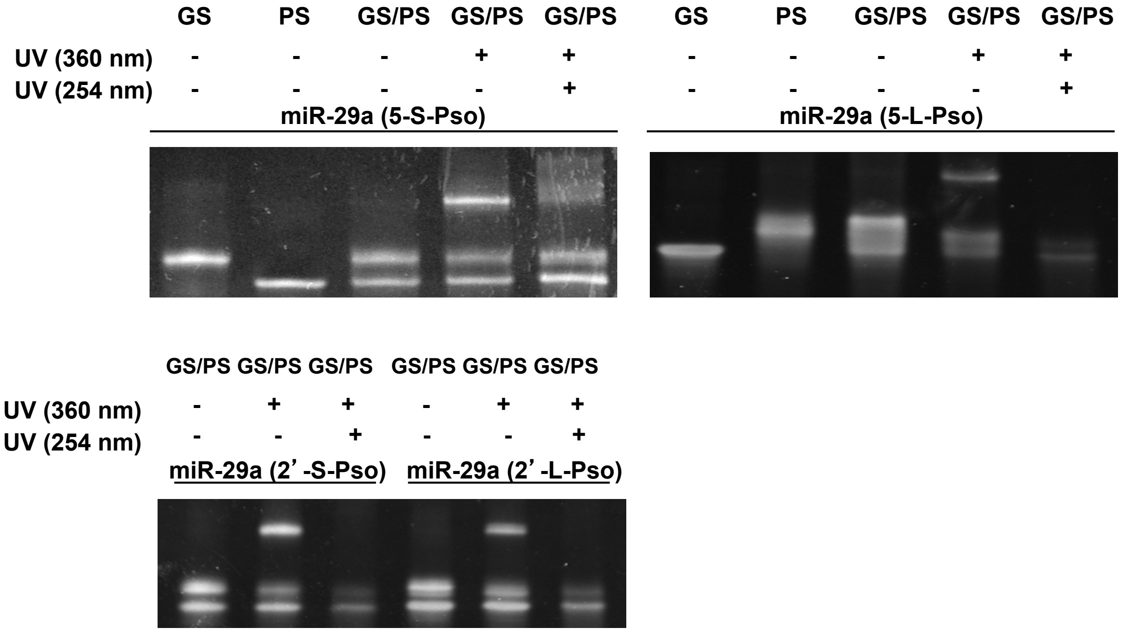

Supplementary Figure 2

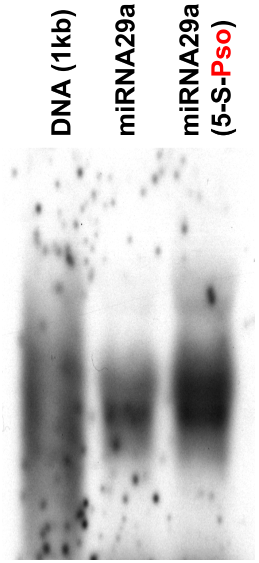

Supplementary Figure 3

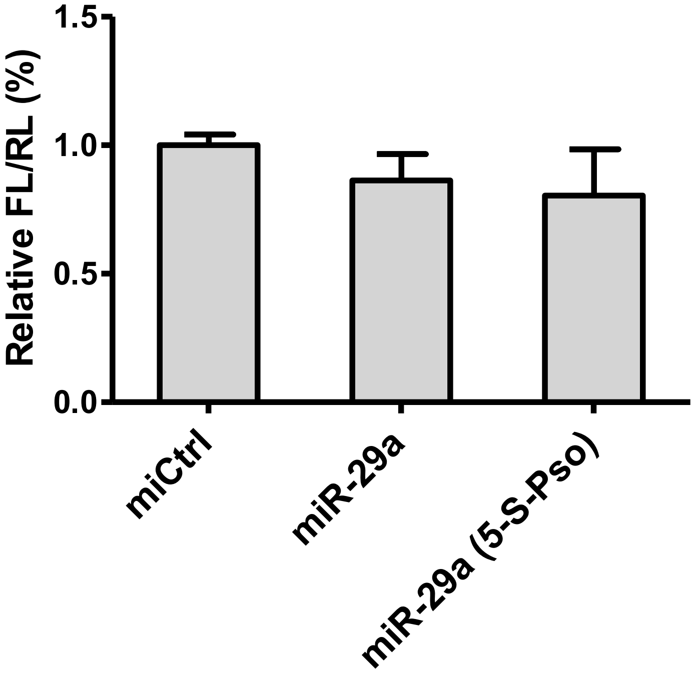

Supplementary Figure 4

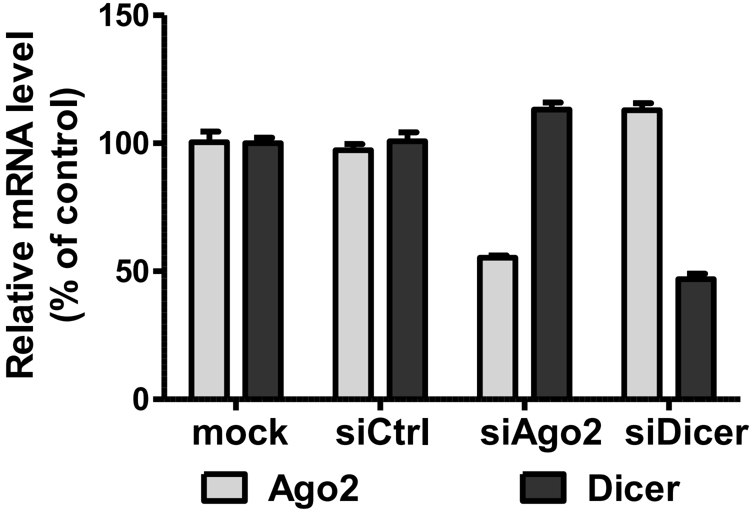

Supplementary Figure 5

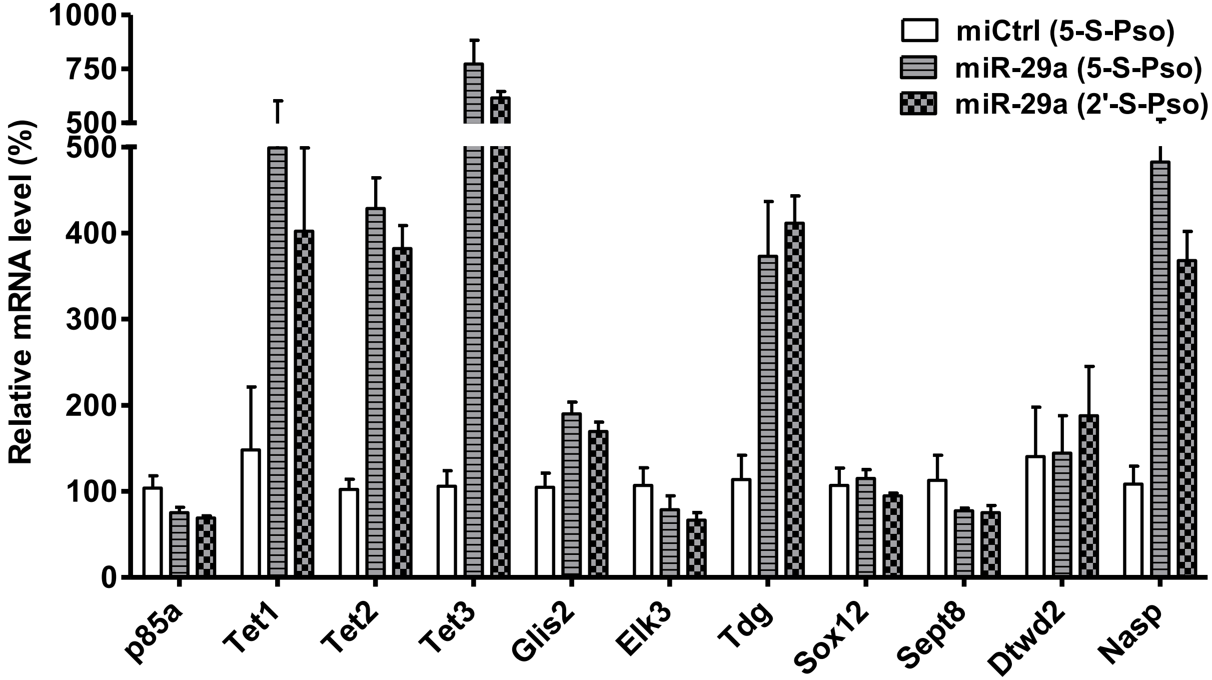

Supplementary Figure 6

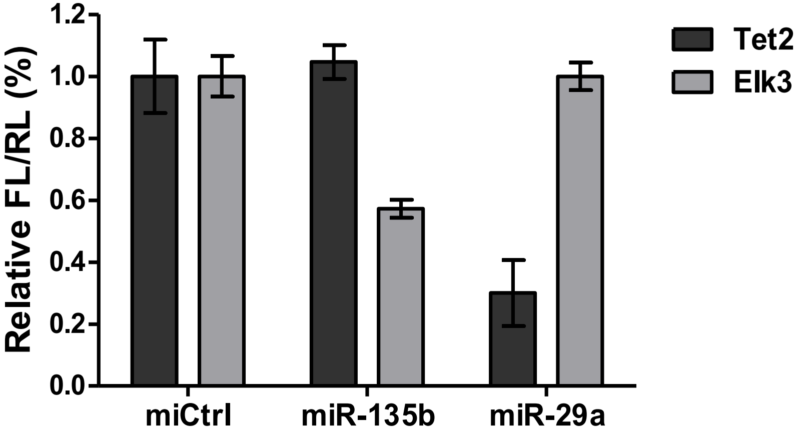

Supplement: Supplementary file 1 [file anie0051-5880-sd1.pdf]
